# Effects of Mealworm‐Based Synbiotics on Productive Performance, Egg Quality, Blood Metabolites, and Gut Health in Laying Japanese Quails

**DOI:** 10.1002/fsn3.71281

**Published:** 2025-11-26

**Authors:** Sosan Mohammadi, Mehran Torki, Maryam Darbemamieh

**Affiliations:** ^1^ Animal Science Department, College of Agriculture and Natural Resources Razi University Kermanshah Iran; ^2^ Department of Plant Protection, College of Agriculture and Natural Resources Razi University Kermanshah Iran

**Keywords:** Japanese quails, mealworm, prebiotic, probiotic, productive performance

## Abstract

This study explored the potential of synbiotics, formulated by combining either *Lactobacillus plantarum* or a commercial probiotic (Parsilact) with 1% mealworm (*Tenebrio molitor,* MW) as a prebiotic, on the productive performance, egg quality, blood chemistry, organ weights, intestinal morphology, and cecal microbiota of laying quails. The study involved 192 22‐week‐old laying quails over 56 days. Productive performance parameters, including feed intake, egg production, egg mass, and feed conversion ratio, were not affected by the treatments (*p* > 0.05). In contrast, synbiotic supplementation significantly improved egg quality, with treated birds producing heavier eggs with a greater yolk proportion (*p* = 0.001 and *p* = 0.004, respectively). Blood analysis revealed higher phosphorus levels in birds fed synbiotic diets (*p* = 0.021) and lower triglyceride concentrations in the Parsilact group compared with the control (*p* = 0.001). Regardless of probiotic source, the inclusion of MW enhanced gut health by increasing cecal *Lactobacillus* counts (*p* = 0.001), while *Escherichia coli* remained unaffected. Overall, the findings indicate that incorporating MW into the diet, particularly in a synbiotic formulation, holds potential for enhancing egg quality and gut microbiota in laying quails without compromising overall performance.

## Graphical abstract

This study evaluated synbiotics combining *Lactobacillus plantarum* or a commercial probiotic (Parsilact) with 1% mealworm ( *Tenebrio molitor* ) meal in laying quails. Synbiotic supplementation did not affect feed intake, egg production, or feed efficiency but significantly improved egg quality, increasing egg weight and yolk proportion. Birds receiving synbiotics also exhibited elevated blood phosphorus levels and higher cecal *Lactobacillus* counts. These findings highlight mealworm‐based synbiotics as promising feed additives to enhance egg quality and gut health in laying quails.

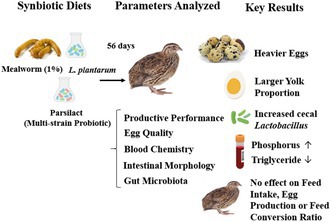

## 1 Introduction

With the rising global demand for protein, quail production is gaining prominence due to its efficient feed conversion, rapid growth, and strong reproductive performance (Ali 2012; Lima et al. 2023). At the same time, growing concerns over antimicrobial resistance and food safety have accelerated the shift toward antibiotic‐free poultry systems (Acosta et al. 2025). Given the restrictions on antibiotic use, functional feed additives such as probiotics, prebiotics, and synbiotics are increasingly explored as natural alternatives to promote gut health, enhance productivity, and reduce antibiotic dependence (Yue et al. 2025).

Promising results have been reported in birds supplemented with probiotics, which are live microorganisms that confer health benefits when administered in adequate amounts (Yang et al. 2024). Previous research indicates that probiotic products based on *Lactobacillus* and *Bacillus* species are the most commonly used in poultry diets (Liang et al. 2021; Sandvang et al. 2021). *Lactobacillus plantarum* ( *L. plantarum* ), a facultative heterofermentative species of the *Lactobacillus* genus, has shown particular effectiveness in enhancing gut health, nutrient digestibility, and overall productive performance (Liang et al. 2021; Yang et al. 2024; Saeed et al. 2025). The literature further suggests that using multiple probiotic species may be more advantageous than single strains, as synergistic interactions among strains can amplify their beneficial effects (Kwoji et al. 2021; Reuben et al. 2022). For example, multi‐strain probiotics have been shown to enhance average daily weight gain, feed conversion ratio (FCR), and feed intake (FI) in poultry, while also reducing mortality associated with health challenges (Sultan et al. 2024).

On the other hand, prebiotics, which are non‐digestible feed components that stimulate the growth of beneficial gut bacteria, are receiving growing attention in poultry nutrition (Yue et al. 2025). Insects represent not only a novel and sustainable source of high‐quality protein and energy but are also recognized for their potential prebiotic effects attributed to their chitin content (Biasato et al. 2023). Among them, yellow mealworm ( *Tenebrio molitor* , MW) is particularly noteworthy, offering high‐quality protein with a lower environmental footprint than soybean meal or fishmeal, while simultaneously providing prebiotic properties capable of modulating gut microbiota (Hong et al. 2020). Previous studies have shown that low MW inclusion levels (1%–5%) can enhance growth performance, improve gut health, and reduce intestinal *Escherichia coli* in Japanese quails, while avoiding the adverse effects seen at higher levels (Islam and Yang 2017; Zadeh et al. 2019; Sedgh‐Gooya, Torki, Darbemamieh, Khamisabadi, and Abdolmohamadi 2021, 2021; Secci et al. 2022).

Some researchers believe that combining probiotics with prebiotics can create a synergistic effect, potentially offering greater benefits than either supplement alone (Tufan and Bolacali 2017). Synbiotics provide both the live microorganisms (probiotics) and the fuel they need to thrive (prebiotics) in the gut (Yue et al. 2025). This can lead to a more robust and stable gut microbiota, potentially enhancing the general well‐being and functioning of the host (Yue et al. 2025). For example, synbiotics containing *Bacillus subtilis* and inulin improved egg production (EP) and egg quality in laying hens (Abdelqader et al. 2013). Most synbiotic strategies use conventional carbohydrate prebiotics primarily for their functional benefits (Yue et al. 2025). In contrast, MW‐based synbiotics provide both fermentable chitin and high‐quality protein, offering a dual nutritional–functional benefit that makes them a unique and underexplored alternative to traditional formulations (Islam and Yang 2017; Biasato et al. 2023).

Despite these promising findings, most research has examined probiotics and insect meals independently, with limited attention to their combined use. In particular, the potential of yellow MW as a prebiotic substrate in combination with specific probiotic strains such as *L. plantarum* or commercial probiotic blends in laying quails remains unexplored. This study aimed to investigate the impact of MW inclusion as a prebiotic, in combination with *L. plantarum* and a commercial probiotic (Parsilact) to form synbiotics, on the productive performance, egg quality, blood parameters, organ weights, intestinal morphology, and selected cecal microbiota of laying Japanese quails.

## 2 Materials and Methods

### 2.1 Ethics Statement

This experiment received approval from Razi University's Animal Ethics Committee in Kermanshah, Iran, ensuring all procedures followed strict animal welfare guidelines (IR.RAZI.REC.1400.091). These protocols adhere to European Union standards for animal protection and feed legislation.

### 2.2 Birds and Dietary Treatments

A total of 192 laying Japanese quail (22 weeks old) were completely randomized design with a 2 × 3 factorial arrangement, evaluating the effects of two dietary levels of MW (0% and 1%) and three probiotic treatments (Parsilact, *L. plantarum*, and a control). Each treatment had eight replicates with four quails each. The inclusion rate of 1% MW was selected to test the prebiotic (chitin‐related) effect of MW as a supplemental ingredient while avoiding major reformulation of the basal diet as an energy or protein replacement. The experiment lasted 56 days. Birds were housed in individual cages (50 × 40 × 50 cm^3^) under controlled environmental conditions (20°C–22°C, 50%–55% relative humidity) with *ad libitum* access to feed and water. Sample size was determined based on expected effect sizes from previous studies to achieve 80% power at α = 0.05. A specific type of bacteria named *L. plantarum* obtained from the Iranian Biological Resource Center (IBRC‐M 11042, Tehran, Iran) was cultured in MRS broth (Vandidaz Co., Tehran, Iran) and incorporated into the feed at a concentration of 4 × 10^9^ CFU/kg. The commercial probiotic Parsilact (Pardis Roshd Mehregan Co. Fars, Iran), containing *Bacillus subtilis, Bacillus licheniformis, Bacillus coagulans, Lactobacillus rhamnosus, Enterococcus faecium*, and *L. plantarum* was added at a rate of 100 g/ton of feed. All diets were formulated to meet the nutritional requirements of quail as specified by the National Research Council (NRC) (1994). Standard procedures outlined by the AOAC International (2005) were used to analyze the content of crude protein, crude fiber, ash, crude fat, calcium, and phosphorus. Table 1 presents the ingredients and the resulting composition of the experimental diets.

**TABLE 1:** Ingredients and calculated composition of the experimental basal diets.

Ingredient	Control (%)	Mealworm (%)
Mealworm	—	1
Corn	51.66	50.99
Soybean meal	36.94	35.49
Soybean oil	2.99	2.99
Wheat bran	0.64	1.79
Dicalcium phosphate	1.10	1.07
Calcium carbonate	2.80	2.80
Oyster	2.83	2.85
DL‐Methionine	0.17	0.16
Sodium chloride	0.32	0.32
Trace‐mineral vitamin premix^a^	0.5	0.5
Threonine	0.05	0.04
*Calculated values (%)*		
Metabolizable energy (Kcal/kg)	2900	2900
Crude protein	20.9	20.9
Ether extract	4.97	5.19
Crude fiber	3.79	3.95
Calcium	2.5	2.5
Available phosphorus	0.35	0.35
Linoleic acid	2.99	2.97
Na	0.15	0.15
Arginine	1.23	1.22
Glycine	0.79	0.79
Serine	0.93	0.93
Histidine	0.50	0.50
Isoleucine	0.78	0.79
Leucine	1.60	1.59
Lysine	1.01	1.00
Methionine	0.45	0.45
Cysteine + Methionine	0.75	0.75
Cysteine	0.30	0.31
Phenylalanine	0.90	0.89
Tyrosine	0.78	0.79
Tryptophan	0.28	0.29
Valine	0.87	0.88
Threonine	0.74	0.74

^a^ Mineral and vitamin supplied per kg of diet: antioxidant, 40,000 mg; biotin, 40 mg; pantothenic acid, 1200 mg; D_3_, 800,000 IU; B_12_, 60 mg; folic acid, 52 mg; K_3_, 800 mg; niacin, 720 mg; pyridoxine, 1200 mg; folic acid, 400 mg; A, 2,600,000 IU; B_2_, 2640 mg; B_3_, 1200 mg; E, 7200 mg; choline chloride, 100,000 mg; Se, 80 mg; Cu, 4000 mg; Fe, 20,000 mg; Mn, 40,000 mg; I, 400 mg; Zn, 33,880 mg.

### 2.3 Preparation of Mealworms

Mealworms were bred on a wheat bran diet (16–18 weeks). To provide essential moisture, mealworms were given fresh fruits like carrots and potatoes. The larvae were harvested just before they turned into pupae to ensure they were fully developed. After removing any impurities, the larvae were kept without food for 2 days to empty their digestive systems. These fresh larvae were then frozen (−21°C for 48 h), and dried in an incubator set at 60°C for 24 h. After drying, the mealworms were ground into a uniform consistency. The powder underwent analysis to ascertain the levels of crude protein, ether extract, crude fiber, crude ash, calcium, and phosphorus (AOAC International 2005). Table 2 shows the amino acid profiles of the larvae. The amino acid composition of MW larvae was determined using an Agilent 1260 Infinity HPLC system (Agilent Technologies, USA) following acid hydrolysis. Approximately 100 mg of dried sample was hydrolyzed with 6 N HCl at 110°C for 24 h in sealed glass tubes under nitrogen. After hydrolysis, the samples were filtered, evaporated to dryness, and reconstituted in 0.1 M sodium acetate buffer (pH 7.2). Separation was performed on a C18 reverse‐phase column (Agilent ZORBAX Eclipse AAA, 4.6 × 150 mm, 5 μm) using a binary mobile phase consisting of solvent A (0.1 M sodium acetate buffer, pH 7.2) and solvent B (acetonitrile:methanol:water, 45:45:10, v/v/v) at a flow rate of 1.0 mL/min. Amino acids were detected using a fluorescence detector set at 340 nm excitation and 450 nm emission after pre‐column derivatization with o‐phthalaldehyde (OPA). Quantification was achieved using calibration curves prepared with an amino acid standard mixture (Sigma‐Aldrich A9906). In addition, the formula (ash‐free acid‐detergent fiber [%]—acid‐detergent insoluble protein [%]) was used to calculate the chitin content (Marono et al. 2015).

**TABLE 2:** Analysis of chemical compounds and amino acids of yellow flour *Tenebrio molitor* larvae powder.

Calculated compositions	DM (%)
Dry matter	97.1
Crude protein	52.61
Crude fiber	7.43
Ether extract	28.3
Crude ash	6.78
Chitin	5.6
Calcium	3.50
Phosphorous	6.60
Lysine	2.75
Methionine	0.67
Cysteine	0.43
Cysteine + Methionine	1.01
Arginine	2.59
Threonine	1.90
Isoleucine	2.77
Leucine	3.93
Valine	2.98
Histidine	1.54
Phenylalanine	1.75
Glycine	2.52
Serine	2.16
Alanine	3.24
Proline	3.23
Aspartic acid	3.97
Glutamic acid	5.81
Ammonia	0.93

### 2.4 Productive Performance

Following a 1‐week acclimation period to the experimental diets, data collection began. Body weight (BW) was measured at the start and end of the study to assess overall growth. Daily FI was determined by subtracting the weight of leftover feed from the amount provided. Each day, the eggs laid in each replicate were counted and weighed (EW) to calculate the average egg weight. At the end of each 4‐week period, egg mass (EM) was calculated by multiplying the average daily egg weight by the number of laying hens. FCR, which is a measure of efficiency, was calculated by dividing the total feed consumed by the total weight of eggs produced. Mortality rates were recorded to adjust the data for any losses during the experiment.

### 2.5 Egg Quality Characteristics

At the end of the trial period (Week 30), six eggs were collected in each sampling period (two eggs per replicate, over three consecutive days). Subsequently, egg quality characteristics including Haugh unit, albumen height, shell percentage, shell thickness, albumen percentage and yolk percentage were calculated. To measure shell thickness, a dial and pipe gauge (Ozaki MFG. Co., Tokyo, Japan) were used and measurements were taken at three defined areas on the egg (air cell, equator, and sharp end). Finally, the average of these three values was calculated. The shell percentage was determined using the individual weight of each egg and the weight of its shell alone. After breaking the eggs and separating the yolk from the albumen, the weight of each was determined as a percentage of the total EW. To calculate the Haugh unit the weight of each quail egg and the height of its albumen (measured by a micrometer (Mitutoyo, 0.01 mm, Japan)) were first determined. Then, the Haugh unit values were calculated for each quail egg using the following formula: Haugh unit = 100 log (H + 7.57–1.7 w^0.37^), where, H is the albumen height in mm, and W is the quail EW in g.

### 2.6 Blood Sample Collection

Upon termination of the experiment at Day 56 (30 weeks of age), two blood samples were collected from each replicate. Blood was collected on ice, centrifuged, and the resulting plasma was stored at −20°C for later analysis. Standard kits were used to measure the concentrations of plasma metabolites, including glucose, total protein, albumin, triglyceride, cholesterol, low‐density lipoprotein (LDL), high‐density lipoprotein (HDL), calcium, and phosphorus (Pars Azmun kits, Tehran, Iran).

### 2.7 Internal Organ Weights

Thirty‐day‐old quails were deprived of food for 2 h. Two birds from each pen were selected based on their average weight, then weighed and taken to the slaughterhouse. The liver, heart, and gizzard were weighed and expressed as a percentage of the bird's live body weight.

### 2.8 Gut Morphology

Following a modification of the method by Iji et al. (2001), intestinal morphology was evaluated. Mid‐sections of the duodenum, jejunum, and ileum, each approximately 5 mm in length, were collected. These samples were submerged in a 10% buffered formalin solution for 72 h, excised, and washed with saline solution. Tissue processing, paraffin wax embedding, and sectioning (6 μm) were then performed using a rotary microtome (Leica RM 2145, GMI Inc., USA). The sections were placed on slides, stained with hematoxylin and eosin, and analyzed under a light microscope to assess morphometric indices. Villus height (measured from the villus‐crypt junction), crypt depth (measured from the villus‐crypt junction to the gland's end), and the villus height to crypt depth ratio were measured using Image Pro Plus v 4.5 software (Media Cybernetics, USA).

### 2.9 Microbiota Measurements

At the experiment's conclusion, two quails per pen were euthanized aseptically. Their caeca contents were immediately collected and transferred to sterile plates in the laboratory. To prepare dilutions, 1 g of sample was weighed and mixed with 9 mL of sterile physiological saline (0.9% NaCl solution) to create a 1:10 dilution (10^−1^). This serial dilution process was repeated until a final dilution of 10^−5^ was achieved. MRS agar (Merck, Germany) was used to cultivate *Lactobacillus* bacteria, while MacConkey agar (Merck, Germany) was used for *E. coli* isolation. Petri dishes containing these media were inoculated with 250 μL of each dilution and incubated at 37°C for 48 and 24 h, respectively. After incubation, colony counts for *Lactobacillus* and *E. coli* were determined. Plates yielding colony counts within the statistically viable range of 30–300 colony‐forming units (CFUs) per plate were selected for quantification to ensure accuracy and avoid the masking effects of overcrowding. The number of CFUs per gram of sample was calculated by multiplying the colony count on each plate by the corresponding dilution factor. All microbiological procedures, including media preparation, plating, incubation, and colony counting, were conducted in a sterile environment within a microbiology hood.

### 2.10 Statistical Analyses

The data was analyzed using a completely randomized design with a 2 × 3 factorial arrangement and the general linear model (GLM) in SAS software (SAS Institute 2015). Tukey's multiple comparison test was used to compare means at a significance level of *p* ≤ 0.05. The following model was employed for the analysis:

$$Y_{\mathrm{ijk}}=\mu +M_{i}+P_{j}+{\mathrm{MP}}_{\mathrm{ij}}+e_{\mathrm{ijk}}$$

In this model, *Y*_ijk_ represents the individual observation, μ is the overall mean, *M*_i_ represents the main effect of MW, *P*_j_ represents the main effect of probiotic, MP_ij_ represents the interaction between MW and probiotic, and *e*_ijk_ represents the experimental error. Cages were used as experimental units for productive performance, while individual birds served as units for all other measured parameters.

The assumptions of the GLM were evaluated before conducting the analysis. The normality of the model residuals was tested with the Shapiro–Wilk test, and the homogeneity of variances was assessed with Levene's test. When these assumptions were not met, log or square‐root transformations were applied to the data. However, as the untransformed data met the assumptions for most variables, transformations were only sparingly used.

## 3 Results

### 3.1 Productive Performance

As presented in Table 3, the experimental treatments involving MW, *L. plantarum* , and Parsilact did not exert a statistically significant influence on the productive performance parameters of Japanese quail, including EP ratio, FI, EM, FCR, and BW (*p* > 0.05).

**TABLE 3:** The effect of adding mealworm powder, *Lactobacillus plantarum* bacteria and commercial probiotics (Parsilact) to the diet on the productive performance of laying quails from 22 to 26, 26 to 30, and 22 to 30 weeks of age.

Treatments		Feed intake (g/day/bird)			Egg production (%)			Feed conversion ratio (g feed/g egg)			Egg mass (g/bird)			Body weight (g)
		22‐26 W	26‐30 W	22‐30 W	22‐26 W	26‐30 W	22‐30 W	22‐26 W	26‐30 W	22‐30 W	22‐26 W	26‐30 W	22‐30 W	30 W
*Mealworm* ^1^														
−		28.12	27.79	27.96	78.58	79.67	79.13	2.93	2.88	2.91	9.54	9.71	9.62	268.5
+		28.57	28.43	28.50	80.23	79.66	79.97	2.91	2.89	2.90	9.81	9.85	9.83	271.9
*p* value		NS	NS	NS	NS	NS	NS	NS	NS	NS	NS	NS	NS	NS
SEM		0.649	0.617	0.628	1.31	1.68	1.29	0.037	0.037	0.305	0.176	0.209	0.165	4.36
*Probiotic*														
−		28.88	28.75	28.81	81.93	79.83	80.88	2.93	2.96	2.94	9.89	9.77	9.83	268.4
Parsilact^2^		28.96	28.66	28.81	79.06	81.99	80.53	2.92	2.86	2.89	9.69	10.05	9.88	265.7
*L. plantarum* ^3^		27.21	26.93	27.07	77.31	77.18	77.24	2.91	2.85	2.88	9.44	9.51	9.48	276.6
*p* value		NS	NS	NS	NS	NS	NS	NS	NS	NS	NS	NS	NS	NS
SEM		0.795	0.756	0.769	1.59	2.061	1.86	0.045	0.045	0.373	0.216	0.256	0.202	5.34
*Interactions*														
Mealworm	Probiotic													
−	—	29.34	29.40	29.37	77.25	81.10	80.97	2.99	3.01	3.00	9.76	9.87	9.81	266.1
−	Parsilact	27.76	27.21	27.48	77.50	78.79	78.15	2.91	2.82	2.87	9.54	9.69	9.62	268.3
−	*L. plantarum*	27.27	26.76	27.02	77.37	79.14	78.26	2.88	2.82	2.85	9.31	9.57	9.44	271.2
+	—	28.42	28.09	28.25	82.98	78.59	80.78	2.84	2.92	2.88	9.01	9.67	9.84	270.6
+	Parsilact	30.17	30.12	30.14	80.63	85.19	82.91	2.92	2.90	2.91	9.85	10.42	10.13	263.1
+	*L. plantarum*	27.15	27.10	27.13	77.24	75.21	76.23	2.96	2.88	2.91	9.58	9.46	9.52	281.9
*p* value		NS	NS	NS	NS	NS	NS	NS	NS	NS	NS	NS	NS	NS
SEM^4^		1.12	1.04	1.07	2.29	2.86	2.23	0.062	0.063	0.052	0.313	0.359	0.289	7.63

*Note:* Within a column, while “NS” denotes non‐significance between the corresponding means (*n* = 8 replicates/treatment, 4 quails/replicate).

^1^ 0% or 1% of mealworm powder.

^2^ Parsilact commercial probiotic (including *Bacillus coagulans, Enterococcus faecium, Bacillus subtilis, Bacillus ligiiformis, Lactobacillus rhamnosus*, and *Lactobacillus plantarum* ) in the amount of 100 g per ton of feed.

^3^ *Lactobacillus plantarum* bacteria at the rate of 4 × 10^9^ per kg of ration.

^4^ Standard error of mean.

### 3.2 Egg Quality Traits

The effect of different experimental treatments on egg quality traits is shown in Table 4. Most egg quality measurements including albumen height, Haugh unit, shell percentage, and shell thickness were not affected by the dietary treatments (*p* > 0.05). However the interaction effect of dietary treatments on EW, yolk and albumen percentage exhibited significant differences. Treatments including Parsilact, MW + Parsilact and MW + *L. plantarum* showed the highest EW, significantly exceeding the *L. plantarum* group but no other treatments (*p* = 0.001). The highest percentage of yolk was observed in MW + Parsilact and MW + *L. plantarum* which had a significant difference with the treatment containing *L. plantarum* alone (*p* = 0.004). Regarding the percentage of albumen, the highest was observed in *L. plantarum* and the lowest in treatment MW + Parsilact (*p* = 0.041).

**TABLE 4:** The effect of adding mealworm powder, *Lactobacillus plantarum* bacteria and commercial probiotics (Parsilact) to the diet on the egg quality traits of laying quails.

Treatments		Egg weight (g)	Albumen (%)	Yolk (%)	Albumen height (mm)	Haugh unit	Shell weight (%)	Shell thickness (mm)
*Mealworm* ^1^								
−		12.28^b^	58.80	32.27	4.77	90.17	8.93	21.56
+		12.58^a^	58.40	32.75	4.73	89.88	8.85	21.58
*p* value		0.026	NS	NS	NS	NS	NS	NS
SEM		0.096	0.196	0.188	0.049	0.267	0.065	0.905
*Probiotic*								
−		12.31	58.44	32.61	4.77	90.29	8.96	21.54
Parsilact^2^		12.63	58.39	32.82	4.79	90.11	8.79	21.64
*L. plantarum* ^3^		12.34	58.98	32.09	4.68	89.69	8.92	21.54
*p* value		NS	NS	NS	NS	NS	NS	NS
SEM		0.118	0.239	0.229	0.061	0.327	0.079	0.111
*Interactions*								
Mealworm	Probiotic							
−	—	12.25^ab^	58.16^ab^	32.60^ab^	4.78	90.46	8.96	21.52
−	Parsilact	12.70^a^	58.77^ab^	32.51^ab^	4.87	90.33	8.72	21.69
−	*L. plantarum*	11.87^b^	59.47^a^	31.41^b^	4.66	89.74	9.12	21.48
+	—	12.37^ab^	58.71^ab^	32.33^ab^	4.77	90.13	8.96	21.56
+	Parsilact	12.56^a^	58.01^b^	33.13^a^	4.72	89.89	8.86	21.59
+	*L. plantarum*	12.82^a^	58.49^ab^	32.79^a^	4.69	89.63	8.73	21.60
*p* value		0.001	0.041	0.004	NS	NS	NS	NS
SEM^4^		0.157	0.331	0.312	0.087	0.467	0.109	0.158

*Note:* Within a column, different letters indicate significant differences (*p* ≤ 0.05), while “NS” denotes non significance between the corresponding means (*n* = 8 replicates/treatment [2 eggs/replicate, measured over 3 days]).

^1^ 0% or 1% of mealworm powder.

^2^ Parsilact commercial probiotic (including *Bacillus coagulans, Enterococcus faecium, Bacillus subtilis, Bacillus ligiiformis, Lactobacillus rhamnosus*, and *Lactobacillus plantarum* ) in the amount of 100 g per ton of feed.

^3^ *Lactobacillus plantarum* bacteria at the rate of 4 × 10^9^ per kg of ration.

^4^ Standard error of mean.

### 3.3 Blood Parameters

Table 5 summarizes the effects of adding MW, *L. plantarum*, and Parsilact to the diet of laying quails on their blood chemistry at 30 weeks of age. Most blood parameters remained unaffected by the dietary treatments (*p* > 0.05). However, significant interaction effects between the dietary treatments were observed for phosphorus and triglyceride concentrations. Specifically, quails fed a combination of MW and Parsilact exhibited the highest blood phosphorus levels, significantly surpassing those fed only MW (*p* = 0.021). Notably, while the MW + Parsilact group exhibited the highest triglyceride concentrations, which were significantly elevated compared to those fed only MW or Parsilact alone, the Parsilact‐only group displayed the lowest triglyceride levels, significantly different from the control diet (*p* = 0.001).

**TABLE 5:** The effect of adding mealworm powder, *Lactobacillus plantarum* bacteria and commercial probiotics (Parsilact) to the diet on the blood biochemical parameters of laying quails.

Treatments		Glucose (Mg/dL)	Protein (Mg/dL)	Cholesterol (Mg/dL)	Albumin (Mg/dL)	Calcium (Mg/dL)	Phosphorous (Mg/dl)	Triglyceride (Mg/dL)	LDL^5^ (Mg/dL)	HDL^6^ (Mg/dL)
*Mealworm* ^1^										
−		265.6	4.39	165.1	1.80	8.33	7.88	75.29	114.6	35.46
+		283.9	4.26	177.3	1.74	8.05	7.98	81.38	127.3	33.73
*p* value		NS	NS	NS	NS	NS	NS	NS	NS	NS
SEM		6.58	0.135	7.37	0.068	0.443	0.468	3.86	6.88	1.51
*Probiotic*										
−		273.9	4.31	167.9	1.75	8.16	6.62^b^	78.32	118.0	34.21
Parsilact^2^		284.1	4.32	173.1	1.79	8.44	8.78^a^	75.88	120.5	37.38
*L. plantarum* ^3^		266.3	4.36	172.8	1.78	7.97	8.39^ab^	80.81	124.4	32.20
*p* value		NS	NS	NS	NS	NS	0.024	NS	NS	NS
SEM		8.06	0.165	9.02	0.083	0.542	0.573	4.73	8.43	1.85
*Interactions*										
Mealworm	Probiotic									
−	—	268.3	4.69	176.8	1.85	9.49	7.61^ab^	85.13^ab^	124.7	35.08
−	Parsilact	277.3	4.09	149.8	1.85	7.43	8.00^ab^	55.38^c^	100.4	38.25
−	*L. plantarum*	251.3	4.41	168.9	1.71	8.06	8.03^ab^	85.38^ab^	118.74	33.06
+	—	279.5	3.94	159.0	1.65	6.83	5.63^b^	71.50^bc^	111.35	33.35
+	Parsilact	290.9	4.54	196.4	1.73	9.45	9.55^a^	96.38^a^	140.59	36.51
+	*L. plantarum*	281.3	4.30	176.6	1.85	7.88	8.76^ab^	76.25^abc^	130.04	31.34
*p* value		NS	NS	NS	NS	NS	0.021	0.001	NS	NS
SEM^4^		11.57	0.221	12.07	0.117	0.696	0.789	4.99	11.48	2.68

*Note:* Within a column, different letters indicate significant differences (*p* ≤ 0.05), while “NS” denotes non‐significance between the corresponding means (*n* = 8 replicates/treatment, 2 quails/replicate).

^1^ 0% or 1% of mealworm powder.

^2^ Parsilact commercial probiotic (including *Bacillus coagulans, Enterococcus faecium, Bacillus subtilis, Bacillus ligiiformis, Lactobacillus rhamnosus*, and *Lactobacillus plantarum* ) in the amount of 100 g per ton of feed.

^3^ *Lactobacillus plantarum* bacteria at the rate of 4 × 10^9^ per kg of ration.

^4^ Standard error of mean.

^5^ Low‐density lipoprotein.

^6^ High‐density lipoprotein.

### 3.4 Internal Organ Weight

The results in Table 6 indicate that the experimental treatments had no noticeable impact on the relative weights of the liver, heart, and gizzard in laying quails (*p* > 0.05).

**TABLE 6:** The effect of adding mealworm powder, *Lactobacillus plantarum* bacteria and commercial probiotics (Parsilact) to the diet on the internal organ weights of laying quails.

Treatments		Live weight (g)	Heart (%)	Liver (%)	Gizzard (%)
*Mealworm* ^1^					
−		271.8	0.719	2.50	1.88
+		274.3	0.778	2.51	2.05
*p* value		NS	NS	NS	NS
SEM		5.19	0.028	0.091	0.069
*Probiotic*					
−		265.4	0.782	2.62	2.05
Parsilact^2^		281.1	0.780	2.38	2.02
*L. plantarum* ^3^		272.7	0.684	2.43	1.82
*p* value		NS	NS	NS	NS
SEM		6.63	0.034	0.111	0.085
*Interactions*					
Mealworm	Probiotic				
−	—	270.9	0.734	2.76	2.04
−	Parsilact	274.7	0.783	2.32	1.90
−	*L. plantarum*	269.8	0.643	2.42	1.69
+	—	259.8	0.830	2.48	2.06
+	Parsilact	287.5	0.778	2.44	2.14
+	*L. plantarum*	275.5	0.725	2.43	1.95
*p* value		NS	NS	NS	NS
SEM^4^		9.01	0.049	0.158	0.121

*Note:* Within a column, while “NS” denotes non significance between the corresponding means (*n* = 8 replicates/treatment, 2 quails/replicate).

^1^ 0% or 1% of mealworm powder.

^2^ Parsilact commercial probiotic (including *Bacillus coagulans, Enterococcus faecium, Bacillus subtilis, Bacillus ligiiformis, Lactobacillus rhamnosus*, and *Lactobacillus plantarum* ) in the amount of 100 g per ton of feed.

^3^ *Lactobacillus plantarum* bacteria at the rate of 4 × 10^9^ per kg of ration.

^4^ Standard error of mean.

### 3.5 Intestinal Morphology

**FIGURE 1:**
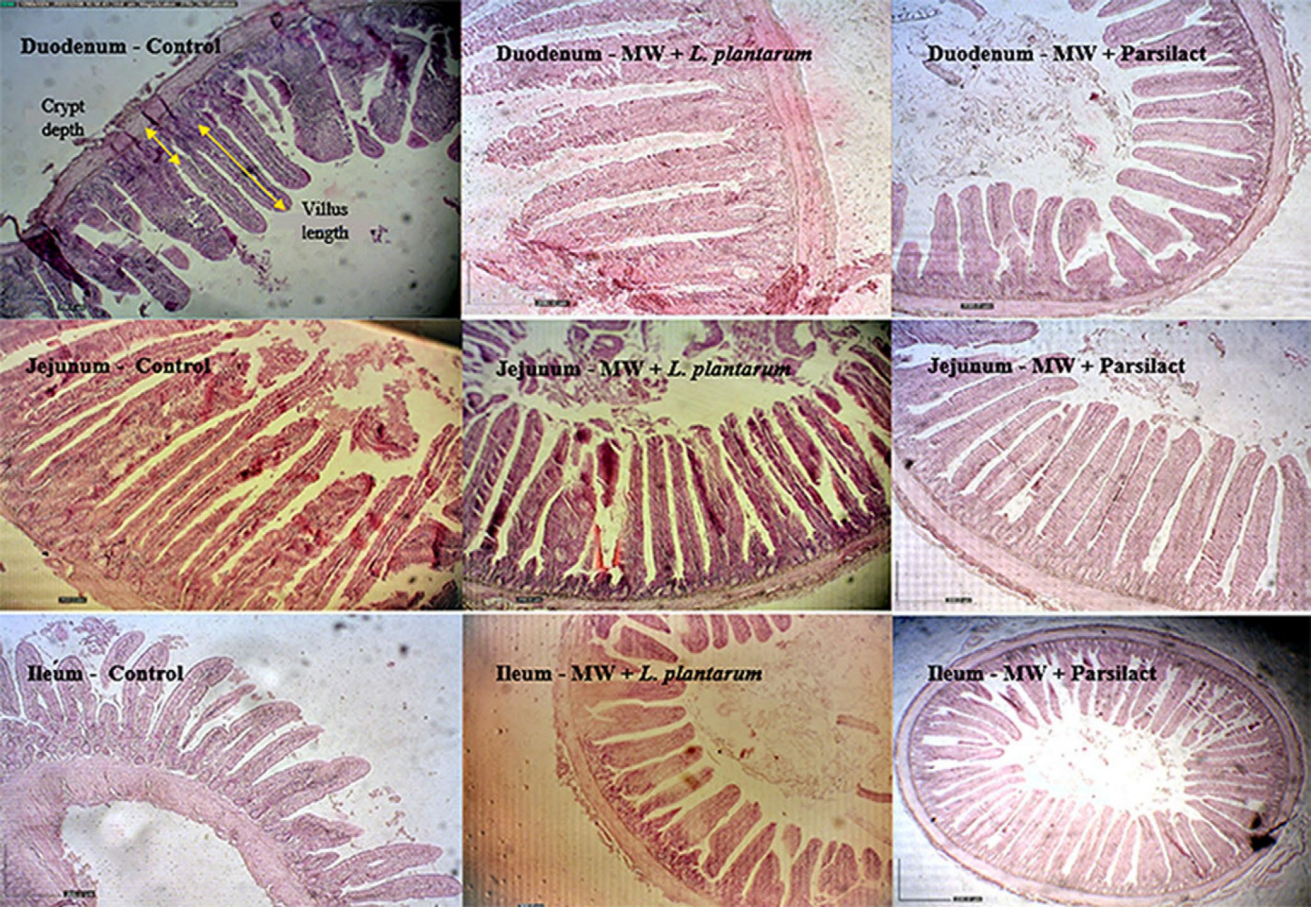
Representative histomorphology of the small intestine in quails fed the control diet or synbiotic treatments (MW + *L. plantarum* and MW + Parsilact). Sections of the duodenum, jejunum, and ileum are shown for each group. Tissues were stained with hematoxylin and eosin. Magnification: 276×; scale bar = 200 μm. Although morphometric measurements in Table 7 showed no significant differences (*p* > 0.05) in villus height, crypt depth, or villus height to crypt depth ratio among treatments, these representative images illustrate tissue integrity and confirm the quality of the analysis.

Table 7 presents the intestinal morphometric measurements. No significant differences (*p* > 0.05) were observed among treatments in villus height, crypt depth, or villus height‐to‐crypt depth ratio in the duodenum, jejunum, or ileum. Figure 1 illustrates representative histology images of the small intestine for the control and synbiotic treatment groups (MW + *L. plantarum* and MW + Parsilact).

**TABLE 7:** The effect of adding mealworm powder, *Lactobacillus plantarum* bacteria and commercial probiotics (Parsilact) to the diet on the intestinal morphology of laying quails.

Treatments		Duodenum			Jejunum			Ileum		
		Villus length (μm)	Crypt depth (μm)	V/C^5^	Villus length (μm)	Crypt depth (μm)	V/C	Villus length (μm)	Crypt depth (μm)	V/C
*Mealworm* ^1^										
−		703.01	114.2	6.37	551.0	90.31	6.82	435.9	91.51	4.97
+		687.80	109.6	6.64	617.4	102.91	6.35	440.4	109.77	4.22
*p* value		NS	NS	NS	NS	NS	NS	NS	NS	NS
SEM		35.69	3.54	0.419	29.04	5.95	0.499	23.56	5.07	0.276
*Probiotic*										
−		680.2	114.6	6.19	616.9	107.1	6.03	415.1	101.2	4.34
Parsilact^2^		671.9	115.2	6.10	587.3	96.91	6.75	458.2	103.5	4.67
*L. plantarum* ^3^		734.1	105.9	7.24	548.4	85.88	6.99	441.2	97.2	4.78
*p* value		NS	NS	NS	NS	NS	NS	NS	NS	NS
SEM		43.72	6.13	0.513	35.56	7.29	0.612	28.86	6.21	0.339
*Interactions*										
Mealworm	Probiotic									
−	—	714.2	121.9	6.11	572.8	111.7	5.41	455.6	90.13	5.21
−	Parsilact	646.2	113.0	5.87	513.9	78.66	6.98	430.1	90.30	5.11
−	*L. plantarum*	748.7	107.6	7.15	566.4	80.61	8.08	421.9	94.14	4.59
+	—	646.3	107.1	6.28	660.9	102.4	6.65	374.5	112.3	3.47
+	Parsilact	697.7	117.3	6.33	660.7	115.2	6.51	486.2	116.8	4.24
+	*L. plantarum*	719.5	104.2	7.32	530.4	91.14	5.89	460.5	100.2	4.97
*p* value		NS	NS	NS	NS	NS	NS	NS	NS	NS
SEM^4^		62.41	8.73	0.739	49.62	10.02	0.849	40.29	8.83	0.466

*Note:* Within a column, while “NS” denotes non‐significance between the corresponding means (*n* = 8 replicates/treatment, 2 quails/replicate).

^1^ 0% or 1% of mealworm powder.

^2^ Parsilact commercial probiotic (including *Bacillus coagulans, Enterococcus faecium, Bacillus subtilis, Bacillus ligiiformis, Lactobacillus rhamnosus*, and *Lactobacillus plantarum* ) in the amount of 100 g per ton of feed.

^3^ *Lactobacillus plantarum* bacteria at the rate of 4 × 10^9^ per kg of ration.

^4^ Standard error of mean.

^5^ Villus height‐to‐crypt depth ratio.

### 3.6 Cecal Microbiota

Table 8 shows the impact of dietary treatments on the microbial populations in the cecum of laying quails. Notably, the interaction effect of treatments significantly influenced the population of *Lactobacillus* bacteria (*p* = 0.001). Quails fed MW exhibited the highest number of *Lactobacillus* compared to all other treatments except the control group. In contrast, the number of *E. coli* bacteria remained unaffected by the dietary interventions (*p* > 0.05).

**TABLE 8:** The effect of adding mealworm powder, *Lactobacillus plantarum* bacteria and commercial probiotics (Parsilact) to the diet on the cecum microbial population of laying quails.

Treatments		*Lactobacillus* (Log10 CFU/g)	*E. coli* (Log10 CFU/g)
*Mealworm* ^1^			
−		7.60	5.09
+		7.59	4.86
*p* value		NS	NS
SEM		0.032	0.144
*Probiotic*			
−		7.81^a^	5.17
Parsilact^2^		7.44^b^	4.83
*L. plantarum* ^3^		7.53^b^	4.93
*p* value		0.001	0.176
SEM		0.040	NS
*Interactions*			
Mealworm	Probiotic		
−	—	7.75^b^	5.39
−	Parsilact	7.43^c^	4.83
−	*L. plantarum*	7.62^bc^	5.06
+	—	7.87^a^	4.95
+	Parsilact	7.45^c^	4.83
+	*L. plantarum*	7.44^c^	4.80
*p* value		0.001	NS
SEM^4^		0.047	0.257

*Note:* Within a column, different letters indicate significant differences (*p* ≤ 0.05), while “NS” denotes non significance between the corresponding means (*n* = 8 replicates/treatment, 2 quails/replicate).

^1^ 0% or 1% of mealworm powder.

^2^ Parsilact commercial probiotic (including *Bacillus coagulans, Enterococcus faecium, Bacillus subtilis, Bacillus ligiiformis, Lactobacillus rhamnosus*, and *Lactobacillus plantarum* ) in the amount of 100 g per ton of feed.

^3^ *Lactobacillus plantarum* bacteria at the rate of 4 × 10^9^ per kg of ration.

^4^ Standard error of mean.

## 4 Discussion

The proximate composition of MW (Table 2) indicates a high crude protein content (52.61%), followed by ether extract (28.3%), crude fiber (7.43%), crude ash (6.78%), and phosphorus (6.60%). These values align with previously reported ranges for MW, which include 47%–60.2% crude protein, 19.1%–36.7% ether extract, and 2.65%–6.99% crude ash (Hong et al. 2020). In contrast, the crude fiber content observed in this study (7.43%) is lower than that reported in studies where MW were reared on specific agricultural by‐products (Kwon et al. 2020; Kröncke and Benning 2023). This variation suggests that feed composition and rearing conditions can substantially influence the nutritional profile of MW.

The present study demonstrated that supplementation with 1% MW, *L. plantarum* , or Parsilact, either individually or in combination, had no significant effect on the productive performance of laying Japanese quails, likely because the birds were already performing near their physiological potential, leaving minimal room for further improvement.

The lack of response to *L. plantarum* aligns with Istiqomah et al. (2020), who also observed no performance changes at a similar inclusion level in quails. In contrast, other studies reported improved EP and FCR with higher doses or multi‐strain probiotics in hens and quails (Manafi et al. 2016; Alaqil et al. 2020; Nour et al. 2021), underscoring that probiotic efficacy depends on strain, dosage, host, and environment. Mechanisms such as pathogen exclusion and immune or gut barrier modulation (Yang et al. 2024) may be dose‐ or strain‐specific and were likely insufficiently activated under the present conditions.

Similarly, the non‐significant effects of MW supplementation are consistent with previous research showing variable outcomes depending on inclusion level and form. Dalle Zotte et al. (2024) observed no improvement in EP or EW with 10% live larvae, whereas lower inclusion levels (≤ 2.5%) have occasionally enhanced laying performance (Hajati and Negarandeh 2021; Sedgh‐Gooya, Torki, Darbemamieh, Khamisabadi, and Abdolmohamadi 2021). The 1% inclusion level used in the present study may therefore have been suboptimal, too low to elicit a measurable prebiotic effect, yet sufficient to slightly alter dietary balance without conferring performance benefits.

The combination of probiotics and MW also failed to produce synergistic or additive effects, consistent with prior studies reporting limited synbiotic benefits when the selected components lack strong functional complementarity (Abdelqader et al. 2013; Youssef et al. 2013). The current findings suggest that either the tested *L. plantarum* strain and MW inclusion level did not interact synergistically, or the basal diet already supported a stable gut microbiota, minimizing potential gains. Reports of improved performance with other probiotic–prebiotic pairings (e.g., *Enterococcus faecium* combined with oligosaccharides; Babazadeh et al. 2011) further emphasize the strain‐ and substrate‐specific nature of such interactions.

The absence of significant differences in albumen height, Haugh unit, shell thickness, and shell percentage among treatments indicates that neither MW inclusion nor probiotic supplementation meaningfully influenced egg external quality. This outcome is consistent with prior research showing minimal response of shell and albumen traits to probiotic or insect meal inclusion in laying quail and hens (Manafi et al. 2016; Saksrithai et al. 2019; Sedgh‐Gooya, Torki, Darbemamieh, Khamisabadi, and Abdolmohamadi 2021).

Interestingly, this study found a slight but significant increase in EW and yolk percentage in synbiotic and Parsilact‐only groups compared with the *L. plantarum* ‐only group. The synergy between MW‐derived chitin and probiotics may have enhanced gut health and nutrient utilization, supporting yolk deposition (Islam and Yang 2017; Biasato et al. 2023; Abenaim and Conti 2025). This aligns with previous reports linking synbiotics to improved intestinal function and nutrient partitioning (Awad et al. 2009; Veldkamp et al. 2012). However, the effect size was small (≈0.3 g) and not different from the control, suggesting limited biological or economic relevance. These results are consistent with earlier studies showing no significant impact of 1%–3% MW on EW (Ko et al. 2020) and modest synbiotic effects in laying hens (Huang et al. 2005; Awad et al. 2009).

The higher albumen percentage in the *L. plantarum* group compared with MW + Parsilact suggests microbial–substrate competition or strain‐specific effects on protein metabolism. Previous studies have shown inconsistent impacts of MW or probiotics on albumen traits (Ko et al. 2020; Nour et al. 2021; Dalle Zotte et al. 2024), indicating dependence on bird age, diet, and additive dosage.

The superior albumen proportion with single‐strain *L. plantarum* implies that targeted microbial activity may support albumen protein synthesis more effectively than multi‐strain formulations. *L. plantarum* enhances magnum secretory function and provides amino acids (e.g., glutamic acid, aspartic acid, threonine, and serine) essential for ovalbumin and ovomucin synthesis (Obianwuna et al. 2022; Saeed et al. 2025). In contrast, Parsilact's mixed strains may involve interspecies competition for these substrates. *Bacillus* spp., particularly *B. subtilis* and *B. licheniformis* , consume considerable amino acids for their own growth (Kambourova et al. 2001; Wang et al. 2022), potentially reducing availability for albumen formation. Thus, the higher albumen percentage in the *L. plantarum* group likely reflects more efficient amino acid utilization toward egg protein synthesis.

The minimal influence of dietary MW, *L. plantarum* , and Parsilact on most blood parameters in laying quails supports previous evidence indicating limited hematological responses to similar interventions (Shariat Zadeh et al. 2020; Sedgh‐Gooya, Torki, Darbemamieh, Khamisabadi, and Abdolmohamadi 2021).

Notably, supplementation with Parsilact alone significantly reduced blood triglyceride levels, aligning with evidence that probiotics can modulate lipid metabolism through bile acid deconjugation and cholesterol conversion pathways (Nopparatmaitree et al. 2022; Hamzehee et al. 2025). The multi‐strain composition of Parsilact, comprising *Bacillus* spp., *Lactobacillus*, and *Enterococcus*, likely exerts complementary effects, including enhanced fatty acid β‐oxidation via activation of peroxisome proliferator‐activated receptor‐α (PPAR‐α) and suppression of hepatic lipogenesis through short‐chain fatty acid (SCFA) production (Kwoji et al. 2021; Nopparatmaitree et al. 2022; Tsai et al. 2023; Hamzehee et al. 2025). Together, these mechanisms contribute to improved lipid utilization and lower circulating triglyceride concentrations. MW supplementation also lowered triglycerides, likely due to chitin binding dietary fats and bile acids, reducing lipid absorption (Yildirim et al. 2020; Abenaim and Conti 2025). However, inconsistent findings in broilers (Biasato et al. 2017, 2018) suggest outcomes depend on insect meal level, processing, and species. Similar variability has been reported for probiotics (Alaqil et al. 2020; Jazi et al. 2020). Unexpectedly, the MW and Parsilact combination increased serum triglycerides, contrary to the typical synergistic hypolipidemic effects of synbiotics (Kwoji et al. 2021; Hamzehee et al. 2025). This may reflect enhanced lipid absorption rather than altered metabolism. The lipid‐rich nature of MW, combined with probiotic‐induced improvements in intestinal enzyme activity and villus structure, could increase fat digestibility and transport (Ravzanaadii et al. 2012; Halder et al. 2024). Additionally, MW‐derived fermentable substrates may have shifted microbial fermentation or SCFA profiles toward lipogenic pathways (Abenaim and Conti 2025). However, to clarify the exact underlying mechanisms, further investigations should examine hepatic lipid metabolism in more detail, including the expression of key regulatory genes and bile acid composition and flux.

A significant increase in blood phosphorus concentration was observed in quails fed the combination of MW and Parsilact compared to those fed MW alone. This finding contrasts with earlier studies reporting no notable effect of probiotics or MW on serum phosphorus (Tang et al. 2017; Shariat Zadeh et al. 2020). Although mealworm larvae are rich in minerals such as phosphorus and calcium, their bioavailability can be limited by chitin and other anti‐nutritional factors (Ravzanaadii et al. 2012; Abenaim and Conti 2025). The elevated phosphorus levels in the MW + Parsilact group likely reflect enhanced mineral utilization resulting from the enzymatic and physiological activities of the multi‐strain probiotic. Specifically, *L. plantarum* lowers intestinal pH to improve mineral solubility (Qiao et al. 2019), while *Bacillus* spp. produce phosphatases and phytases that release bound phosphorus (Luise et al. 2022). *B. licheniformis* and *E. faecium* further enhance mineral absorption and nutrient digestibility through improved gut morphology and enzyme production (Li et al. 2022; Cao et al. 2024). Additionally, the production of SCFAs by these microbes promotes epithelial proliferation, increasing intestinal surface area and facilitating nutrient uptake (Kwoji et al. 2021). We acknowledge that the proposed mechanisms for higher blood phosphorus in the MW + Parsilact group are speculative. Future studies using phosphorus digestibility assays and analysis of intestinal phosphorus transporter expression (e.g., NaPi‐IIb) are needed to directly confirm the role of probiotics in enhancing mineral utilization.

Consistent with our findings, the relative weights of the liver, heart, and gizzard were not influenced by MW inclusion, aligning with previous reports in quails and broilers (Islam and Yang 2017; Zadeh et al. 2019; Yildirim et al. 2020). Similarly, synbiotic supplementation has been shown to exert no effect on internal organ weights in laying quails (Tufan and Bolacali 2017). Only Ballitoc and Sun (2013) reported increased gizzard weight in broilers fed MW, suggesting that responses may depend on bird species, diet composition, or MW processing method.

The absence of changes in intestinal morphology aligns with the findings of Sedgh‐Gooya et al. (2022), who observed no morphological alterations in laying hens fed varying MW levels. However, previous studies show mixed responses to probiotics and MW. Certain *Lactobacillus* strains have been associated with increased villus height and improved nutrient absorption (Siadati et al. 2018; Shah et al. 2019), whereas *B. subtilis* supplementation has been linked to reduced villus‐to‐crypt ratios in quails (Manafi et al. 2016), highlighting strain‐dependent effects. Similarly, MW has been reported to either enhance (Zadeh et al. 2019) or not affect gut morphology (Biasato et al. 2017, 2018). Such inconsistencies likely reflect differences in MW composition, probiotic strain, dosage, bird species, and age.

The absence of significant effects on *E. coli* populations agrees with previous reports showing that insect‐based diets, even at higher MW inclusion levels, do not alter *E. coli* abundance in laying birds (Hajati and Negarandeh 2021; Stastnik et al. 2021). The 1% MW level used here was likely insufficient to affect this parameter. In contrast, MW supplementation increased *Lactobacillus* counts, likely due to chitin acting as a prebiotic substrate (Khempaka et al. 2011). Unexpectedly, synbiotic treatments (MW + *L. plantarum* or Parsilact) and Parsilact alone showed lower *Lactobacillus* abundance, suggesting possible negative interactions between MW components and probiotic strains. Similar inconsistencies have been reported where MW–probiotic combinations did not enhance *Lactobacillus* growth despite reducing *E. coli* levels (Islam and Yang 2017). These findings indicate that MW–probiotic interactions may not always be synergistic and highlight the need for further research, using approaches such as metagenomic sequencing or in vitro co‐culture with MW metabolites, to clarify the underlying mechanisms.

## 5 Conclusion

The present study demonstrated that dietary supplementation with MW, *L. plantarum* , and Parsilact did not significantly affect productive performance traits (FI, EP, EM, FCR, and BW), internal organ weights, intestinal morphology, or most egg quality parameters in Japanese quails. However, some specific responses were observed. Quails receiving diets containing Parsilact, as well as the combinations of MW with either Parsilact or *L. plantarum* , produced eggs with higher average EW and yolk percentage compared to certain other treatments. In terms of blood chemistry, MW combined with Parsilact increased phosphorus and triglyceride concentrations, whereas Parsilact alone was associated with the lowest triglyceride levels relative to the control. Furthermore, MW supplementation promoted a higher abundance of *Lactobacillus* in the cecum, although no effect was detected on *E. coli* . Overall, these findings suggest that while MW, *L. plantarum* , and Parsilact did not alter general performance or most physiological parameters, they may influence selected egg quality traits, specific blood metabolites, and cecal *Lactobacillus* populations. It should be noted that only a single MW inclusion level (1%) was tested, which may limit the generalizability of the findings. Future studies using multiple inclusion levels, longer experimental durations, and larger sample sizes are warranted to clarify potential dose‐dependent effects and optimize synbiotic formulations for quails.

## Author Contributions

**Sosan Mohammadi:** writing – original draft, investigation, methodology, software, data curation, and formal analysis. **Mehran Torki:** writing – review and editing, conceptualization, supervision, and funding acquisition, investigation. **Maryam Darbemamieh:** investigation, resources, software, and validation.

## Funding

The authors have nothing to report.

## Conflicts of Interest

The authors declare no conflicts of interest.

## Data Availability

The data that support the findings of this study are available from the corresponding author upon reasonable request.
